# Energy recovery from tubular microbial electrolysis cell with stainless steel mesh as cathode

**DOI:** 10.1098/rsos.170967

**Published:** 2017-12-20

**Authors:** Xiaoli Ma, Zhifeng Li, Aijuan Zhou, Xiuping Yue

**Affiliations:** 1 College of Environmental Science and Engineering, Taiyuan University of Technology, Taiyuan 030024, China; 2 Xinneng Nuclear Engineering Co., Ltd of CNNC, Taiyuan 030024, China

**Keywords:** microbial electrolysis cell, stainless steel mesh, columbic efficiency, energy recovery

## Abstract

In comparison to the transportation and storage of hydrogen, methane has advantages in the practical application, while the emerging product termed as ‘biohythane’ could be an alternative to pure hydrogen or methane in a new form of energy recovery from microbial electrolysis cell (MEC). However, the cathodic catalyst even for biohythane still bothers the performance and cost of total MEC. Herein, we fabricated the MEC reactor with surrounding stainless steel mesh (SSM) to investigate the feasibility of stainless steel mesh as an alternative to precious metal in biohythane production. The columbic efficiency (CE) of anode was around at 80%, representing the SSM would not limit the activity of anodic biofilm; the SEM image and ATP results accordingly indicated the anodic biofilm was mature and well constructed. The main contribution of methanogens that quantified by qPCR belonged to the hydrogenotrophic group (*Methanobacteriales*) at cathode. The energy efficiency reached more than 100%, reached up to approximately 150%, potentially suggesting the energetic feasibility of the application to obtain biohythane with SSM in scale-up MEC. Benefiting from the likely tubular configuration, the ohmic resistance of cathode was very low, while the main limitation associated with charge transfer was mainly caused by biofilm formation. The total performances of SSM used in the tubular configuration for biohythane production provide an insight into the implementation of non-precious metal in future scale-up pilot with energy recovery.

## 1. Introduction

Energy crisis was a potential threat for human advance as the depletion of fossil fuel occurred at a high rate and was unrenewable and unsustainable. Biohydrogen production by dark- and photo-fermentation provided a new way that was clean and effective to solve this threat [1]. As an innovative way to convert organic matter to electrons at anode and produce hydrogen at cathode, microbial electrolysis cell have attracted more and more attention [2]. Microbial electrolysis cell (MEC) was a breakthrough for dark-fermentation with glucose as it can use volatile fatty acid (VFA) for hydrogen evolution. More and more studies focused on factors in terms of electrode spacing [3], catholyte [4], anode microbial community [5], ion transport resistance [6], buffer solution [7], substrate [8] etc. have enhanced the performance of hydrogen production of microbial electrolysis cell. Under the condition that performance of microbial electrolysis cell has been enhanced significantly, more modified configurations adapted to the scale-up tests need to be done to evaluate the performance of MEC in applications.

Among, the material of cathode was a restriction for MEC application, especially for Pt/C that was a kind of precious metal with expensive price. Hence, many studies have concentrated on searching for low-cost and high-performance alternative material for MEC application [9,10]. Nickel foam (NF), stainless steel wool (SSW), platinum coated stainless steel mesh (Pt), and molybdenum disulfide coated stainless steel mesh (MoS_2_) electrodes were assessed at different initial pHs using unbuffered catholytes in microbial electrolysis cells [11]. Recently, stainless steel mesh (SSM) was widely applied for microbial electrolysis cell as alternative cathode catalyst [12], because of its low cost [13] and high surface area [14].

Another inevitable obstacle that was methane having been evolved in single microbial electrolysis cell (SMEC) as end product limited further application of MEC for biohydrogen. Methane was detected only in the glucose-fed microbial fuel cell (MFC) rather than acetate type, which means that anode respiring bacteria (ARB) could out-compete acetoclastic methanogens [15]. Wang *et al*. [16] deemed that methane production was related to applied voltage, while there is no significant relevance with methanogen. Cheng *et al*. [17] supported the theory that electromethanogenesis enhanced methane production at cathode. Methane also may be synthesised via converting CO_2_ with proton and electron at cathode, according to the electrosynthesis equation:

$$\text{C}{\text{O}}_{2}+8{\text{H}}^{+}+8{\text{e}}^{-}=\text{C}{\text{H}}_{4}+2{\text{H}}_{2}\text{O}.$$

This testified that methane can be produced from electrosynthesis as CO_2_ was trapped in SMEC at cathode [18,19]. Considering the multiple pathways for methane generation, as well as the methane, which takes advantages in the safety of transportation and storage, could be an alternative sustainable energy to hydrogen. Moreover, the biohythane was becoming a better choice to be produced in MEC. However, although the recent focuses have been transferred from hydrogen recovery to achieve biohythane generation, the proper cathode material that potentially adapted to practical implication is scarcely insufficient.

Considering MEC is a promising assisted strategy to enhance energy recovery from wastes; however, the insufficient investigations related to cathodic catalysts that predominantly determines the cost for practical application restricted the development of it. Herein, a new reactor was constructed with circled stainless steel mesh as cathode, we aimed to evaluate the performances of MEC shelled with stainless steel mesh, in terms of biohythane production, anodic bio-activity and cathodic resistance.

## 2. Method and material

### 2.1. Single microbial electrolysis cell construction

Biohydrogen process was conducted using a SMEC that was made of polycarbonate as previously described [20]. A single cube within a cylinder chamber 7 cm long by 5 cm in diameter (empty bed volume of 137 ml). The heated graphite brush (25 mm diameter, 25 mm length; 0.22 m^2^ surface area; fibre type: PANEX 33 160 K, ZOLTEK) which was soaked in acetone for 24 h and heated in a muffle furnace at 450°C for 30 min was placed in SMEC as anode [21]. The stainless mesh surrounded graphite brush used as cathode in reactor. Its length is 16 cm and width is 5 cm. The gas flowed via a cylinder syringe (volume of 10 ml) and collected into a gas bag (0.5 l capacity; cali-5-bond, Calibrated Instruments).

### 2.2. Microbial electrolysis cell start-up

Three SMECs were constructed and inoculated with waste activated sludge (WAS) from wastewater treatment plant (WWTP) in Harbin. All reactors were supplied a fixed voltage of 0.6 V (Switching Power Supply, FDPS-150, Fudantianxin Inc., China). The SMECs were fed a 120 ml 50 : 50 mixture of the sodium acetate mixed with 50 mM PBS. Once a reactor produced voltage greater than 0.100 V during a fed-batch cycle, the inoculum was omitted. To obtain the similar performance (current, gas production), re-inoculation was conducted in some reactors. All SMECs were operated under 48 h-batch model in room temperature at 22 ± 2°C.

### 2.3. Biological measurements

The scanning electron microscope (SEM) was observed by the field-emission SEM (FE-SEM), which was made by Hitachi, Japan (model: SU8020). Adenosine triphosphate (ATP) was determined by the BacTiter-GloTM Microbial Cell Viability Assay (G8231, Promega Corporation, Dübendorf, CH) and a GloMax 20/20 Luminometer (Turner BioSystems, Sunnyvale, CA, USA). We followed the description of the manufacturer's protocol for this product. We collected 2 ml of the supernatant from each reactor, and 50 µl ATP reagent to prepare to heat at 38°C for 1 min. We mixed final 500 µl sample with 50 µl ATP reagent to settle in the heating block for 20 s at 38°C. The luminescence was subsequently measured, which was expressed in relative light units (RLU). We used conversion factor of 1.75 × 10^−10^ nmol ATP per cell to transfer the RLU to the number of cell. Quantitative PCR (qPCR) was employed to analyse the abundances of total archaea, total bacteria, *Methanobacteriales*, *Methanomicrobiales*, *Methanosarcinaceae*, and *Methanosaetaceae* for four samples collected from cathode at low and high current respectively [22]. All primers and the process description were shown in the electronic supplementary material, table S1.

### 2.4. Analysis and calculation

Voltage was recorded using a multimeter (model 2700; Keithley Instruments). Gases (hydrogen, carbon dioxide, methane) were measured by a gas chromatograph (Fuli GC9790II, Zhejiang analytical instrument Inc., China). The VFAs in the effluent were analysed using a gas chromatograph (Agilent, 4890D; J&W Scientific, USA) with a flame ionization detector (FID) and an appropriate column (19095N-123 HP-INNOWAX, 30 m × 0.530 mm × 1.00 mm, J&W Scientific, USA) using a nitrogen carrier gas [23,24]. Total chemical oxygen demand (COD) was measured following standard methods (method 5220). The pH was measured using a pH meter (PHS-3C, Yangguang Lab. App. Co., Ltd). Reducing sugars were quantified by 3,5-dinitrosalicylic acid (DNS) colorimetric using a spectrophotometer (DU800, Beckman). Electrochemical impedance spectroscopy (EIS) was employed to investigate the resistance of cathode, which was tested by electrochemical station (CHI660, EX, US) [25]. We used ZSimp software to analyse the results and simulate the spectra. The coulombic efficiency was the coulombic ratio of current to the substrate, the cathode electron recovery represents the coulombs of hydrogen compared to the total coulombs of current. The energy efficiency was the ratio of heat energy in the final products (biohythane) to the input electric energy.

## 3. Results and discussion

### 3.1. Start-up and operation period of single microbial electrolysis cell

Three single microbial electrolysis cell reactors were constructed and operated in 48 h-batch model to obtain similar performance with acetate as solely carbon source. In order to achieve similar coulombic efficiency (CE), some reactors were duplicately inoculated here. During the start-up period, the value of CE was up to nearly 160% and that was similar between different reactors. 1# reactor achieved highest value of 156%. The rest of reactors, 2#, 3#, reached 149% and 152%, respectively. The higher coulombic efficiency in single microbial electrolysis cell could be attributed to hydrogen recycle, in which consumption of hydrogen at anode by anode respiration bacteria have been testified, and this will be a restriction of SMEC advance. Thus methane has been considered as end-product in SMEC for it can prevent extra energy consumption from hydrogen recycled [26].

**Figure 1.**
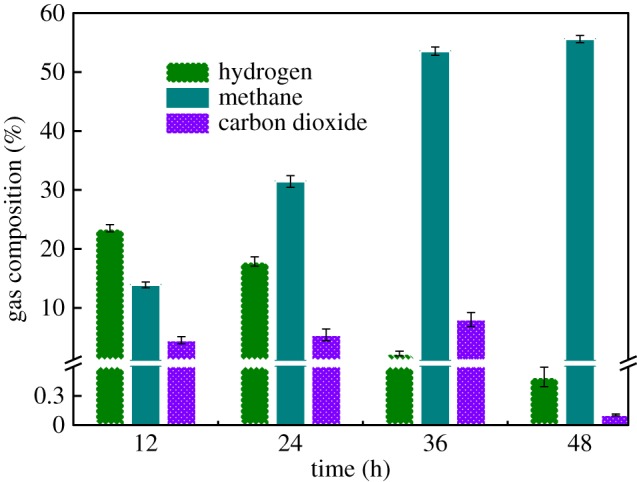
Proportion of biogas.

After the start-up period, as shown in figure 1, hydrogen was the main product for initial few days. Then, methane generated and increased gradually until remaining constant in subsequent 48 h-batch. The proportion of methane in the content of gases that were produced from SMEC went up from 13% to 55%, which represented the content of methane increased as longer operation period and the depletion of hydrogen. The portion of hydrogen that is depleted during the operation period is not corresponding to the content of additional of methane. Hydrogen is not the sole source, however, to transform into methane by hydrogenotrophic methanogens or anode bacteria that can convert H_2_ and CO_2_ to acetate firstly; electrosynthesis of methane also may play an important role in that part. The initial percentage of methane was 13%, representing methane synthesized when hydrogen just evolved; this also supported that hydrogen was not the sole substrate for methane because the time was too short for methanogens growth.

### 3.2. Evaluation on reactor performance

**Figure 2.**
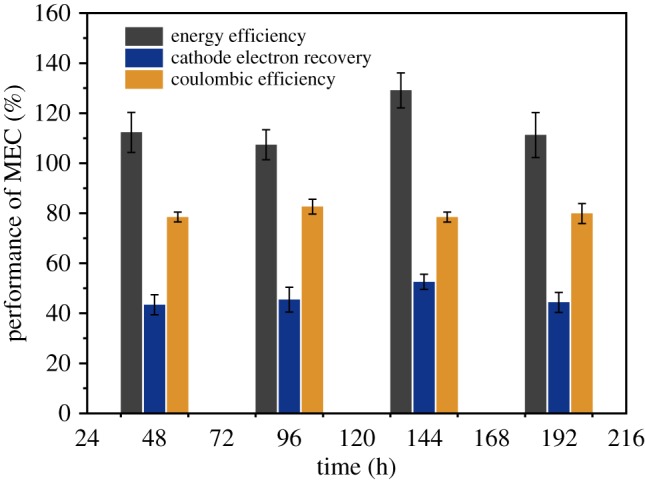
Performance of MEC reactors.

During the stable operation period, as shown in figure 2, in addition to the hydrogen or methane production, the coulombic efficiency could reveal the microbial activity of anode. The coulombic fluctuated around 80%, where the average value was 79.9 ± 2%; this could be ascribed to the methane evolution which avoided the hydrogen recycle compared to higher value in start-up period. Although previous studies showed yield coefficients of f_s_^0^ was 0.05 for *Geobacter sulfurreducens* [27], which was considered as predominant bacteria responsible for extracellular electron transfer, considering the multiple trophic level in anodic biofilm in the presence of syntrophic fermenters which possess higher coefficient [28], moreover, the potential loss should be one of contributors, the coulombic efficiency around 80% was reasonable and satisfactory. Comparing with more than 100% values during start-up period, the phenomenon of hydrogen recycle would be gradually evitable as the growth of methane generation, which was favourable to decrease the ohmic loss that could be ascribed to hydrogen recycle. Another important indication behind the higher coulombic efficiency suggested the modified cathode could be useful to expand the ability of anode without the impediment of the cathodic reaction rate. The cathode electron recovery was of importance to evaluate the performance of catalytic efficiency. As the intrinsic electrochemical property of stainless steel mesh, the electron recovery was low around at 50%; however, the growth of methanogens that had been proved in previous publications could consume electrons for biomass synthesis; moreover, other autotrophic bacteria that could be cooperator with methane generation also led to the loss of electrons. Obviously, it was inevitable for microbial growth in multi-scale application; however, the bio-affinity of cathode also could be enhanced in view of the main contributed methane by microbes. Although the electrochemical methane generation was commonly reported, the strong binding energy of CO was essential, which limited the range of transient metals; copper would be a better choice rather than iron [29,30]. Therefore, the methanogens commonly functioned the duty for methane production from MEC. The modification in the surface of non-precious with carbon materials would be proper to enhance methane generation [31]. However, the lower cathode electron transfer was reasonable if considering the effect of biofilm. Importantly, it seemed that the surrounding structure has boosted the anodic ability in transferring electrons to the electrode without limitation of cathodic reaction rate. This configuration tried to solve the distance between anode and cathode in stimulation of scale-up applications, where the long-range transportation of ionic migration would be the main loss of energy. The positive achievement in energy efficiency would be appreciated for economic feasibility. The energy efficiency stayed beyond the bottom line of 100%, which showed the economic feasibility of this configuration. Although the heat value of methane was lower than hydrogen, while the energy efficiency exceeding 100% represented methane recovery also achieved positive evaluation in energy input and output. Additionally, the finally collected biogas was composed of hydrogen and methane; the biohythane (hydrogen accounted for 12.3 in mixture of hydrogen and methane) was thermodynamically favourable during combustion process that greatly enhanced the methane powered vehicles [32]. Apparently, the biohythane would be a better choice to pure hydrogen or methane in MEC in future application.

### 3.3. Anodic biofilm

**Figure 3.**
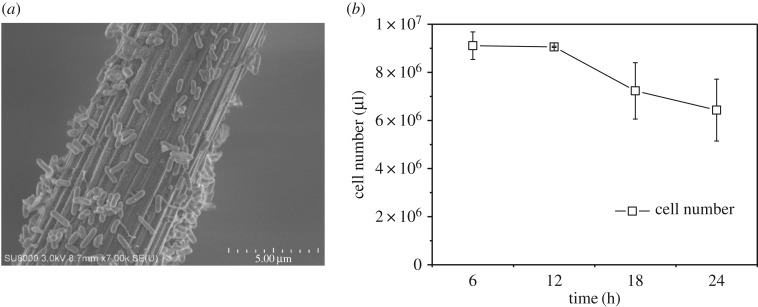
(*a*) SEM images of anodic biofilm. (*b*) Cell number of anodic biofilm.

The biofilm at anode, which drives the electron transfer from organics to electrode, was observed with FE-SEM, as shown in the SEM image (figure 3*a*). The microbes attached on the carbon brush closely, the rod bacteria seemed like physical morphology of *Geobacter*. Although the electron transfer pathway for anode biofilm has been clearly elucidated, consisting of pili-mediated, electron shuttles, and cytochrome. The electron shuttles were soluble and diffused in the substrate. The cytochrome generally attached on the membrane, which took responsibility for electron transfer through physical contact. Both of them were hard to observe directly. The pili, which owned the filament structure, was potentially easier to see. Although some literature reported the observed pili through SEM, it was the far distant view of it, because the real diameter of single nanowire was 4.0 × 10^−9^ m [33], which was invisible with the SEM. In sum, the SEM image not only provided the physical morphology of bacteria attached on the electrode, but also revealed the robust connecting information of bacteria and electrode. Herein, the SEM image showed the anodic biofilm was successfully formed on the electrode.

Besides, the SEM only provided the morphology of biofilm, which failed to give the quantitative details. The ATP detection successfully quantified the cell numbers of bacteria (as shown in figure 3*b*). The variation of ATP in the suspension was accordingly similar to the change of current. The initial high ATP value indicated the rapid response of bacteria to the change of substrate, while the error bar could be influenced by the residue oxygen in the influent. After several hours, the ATP value implied the stable microbial activity. As the decrease in current, the ATP value dropped slightly, which could be caused by the consumption of acetate.

### 3.4. Cathodic electrochemical property

**Figure 4.**
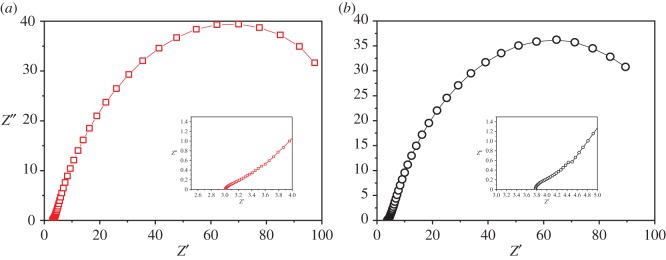
EIS results at low current (*a*) and high current (*b*).

The resistance of electrode determined the energy loss, the EIS detection generally revealed the ohmic resistance, charge transfer and diffusion [34]. Herein, a sine sigmoidal wave was applied to the cell ranging from 100 000 to 0.1 Hz to evaluate the performance of cathode. In order to timely investigate the variation of resistance, the measurement was completed in different current value, which represented the working status of MEC. The measurement was completed at 6.9 mA (figure 4*b*) prior to the current decrease to 1.7 mA (figure 4*a*), where was the second point. According to the results of EIS, two semicircles were obtained during the test, where the first could be ascribed to the electron transport on conductive skeleton, and the second could be caused by the charge transfer. There was no obvious difference in ohmic resistance for lower or higher current value. The inner resistance for low current was 3.667 Ω, and that for high current was 2.994 Ω (as shown in electronic supplementary material, figures S1 and S2 and table S2). However, the charge transfer resistance of higher current was significantly lower than that of lower current (93.48 Ω versus 120.6 Ω).

The ohmic resistance and electron transport resistance should be mainly related with the property of cathode, where the stainless steel mesh, as a kind of transient metal, owns good performance in conductivity, thereby the ohmic resistance was low. The charge transfer was controlled by the chemical reaction rate in the inter-surface of liquid and electrode, a sharp decrease could be regulated by the activity of microbes. The formation of biofilm, which covered the active sites for catalyst in the cathode, dominated the reaction that occurred at the cathode, thus, controlled the charge transfer directly. The decrease could be caused by the variation of microbial activity. Obviously, at initial hours, the high current could be supported by the hydrogen evolution as the microbial community for methane production is unmatured. Once the methane proportion increased, that implied mature community would improve the methane generation to increase the charge transfer resistance with low current.

### 3.5. Cathodic methanogens

**Figure 5.**
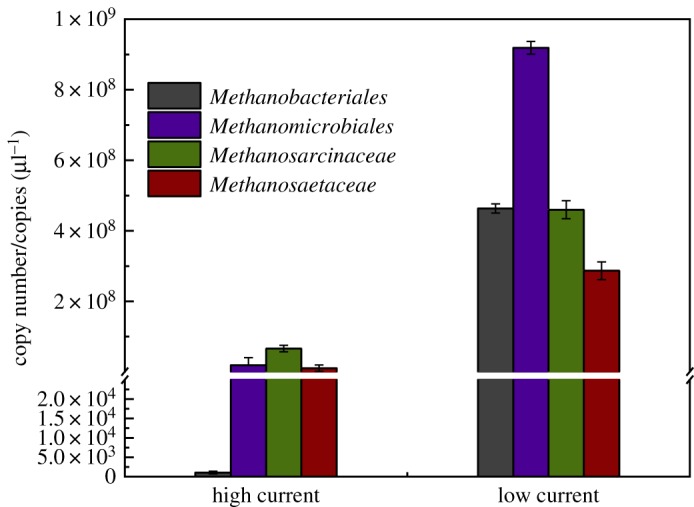
Quantitative analysis of methanogens.

Figure 5 reveals the significant difference of 16S rRNA gene copies of two hydrogenotrophic methanogens orders and two acetoclastic methanogens families in cathodic samples. The numbers of the *Methanobacteriales* and *Methanomicrobiales* 16S rRNA gene which presented by hydrogenotrophic methanogens significantly dominated. The number of *Methanobacteriales* in the cathode biofilm was highest compared with other methanogens (4.6 × 10^8^ copies µl^−1^), which was accordant to the result of Illumina sequencing analysis. *Methanomicrobiales* which represented hydrogenotrophic methanogens was also enriched at the cathode based on the abundant 16S rRNA gene copies (9.2 × 10^7^ copies µl^−1^). However, there was a clear low number of the acetoclastic methanogens contains *Methanosarcinaceae* and *Methanosaetaceae*. At initial hours of operation, the number of methanogens stays low, there is a significant increase as the current decreases in the end of operation period. Not only the hydrogenotrophic methanogens increased significantly, the acetotrophic methanogens also enriched, which is similar to previous studies that proved the two pathways for methane generation by co-effort of hydrogenotrophic and acetoclastic methanogens [35,36]. Obviously, the hydrogenotrophic pathway should be the main contribution to the methane production. The variation of methanogens accordingly verified the charge transfer could be regulated by microbial biofilm at cathode.

## 4. Conclusion

The performance of MEC with cycling cathode proved the non-precious metal could be an alternative to precious one. Besides, the CE and activity of anodic biofilm implied that there were no obvious negative influences caused by the utilization of SSM. Hence, the conclusion from the evaluation of SSM was that it is feasible for MEC to produce biohythane at low cost benefiting from SSM application.

## Supplementary Material

The supplementary showed the specific method of qPCR and the analysis of EIS

## Data accessibility

We include all the experimental data in the electronic supplementary material.
