# Supplementary material for: Energy recovery from tubular microbial electrolysis cell with stainless steel mesh as cathode
Source: R Soc Open Sci. 2017 Dec 20;4(12):170967. doi: 10.1098/rsos.170967 (PMC5750004; doi:10.1098/rsos.170967)
Supplement: The supplementary showed the specific method of qPCR and the analysis of EIS [file rsos170967supp1.docx]

Energy recovery from tubular microbial electrolysis cell with stainless steel mesh as cathode

Xiaoli Ma^1^, Zhifeng Li^2^, Aijuan Zhou^1^, Xiuping Yue^1*^

1 College of Environmental Science and Engineering, Taiyuan University of Technology, Taiyuan 030024, China

2 Xinneng Nuclear Engineering CO., LTD. of CNNC, Taiyuan 030012, China

*Corresponding author: E-mail: environment2016@foxmail.com (Xiuping Yue)

The reaction mixture of 20μL was prepared as the description of SGExcel FastSYBR Mixture(Sangon Biotech (Shanghai) Co.,Ltd), the 2×SGExcel FastSYBR Mixture contains Fast Taq DNA Polymerase、PCR Buffer、dNTPs、SYBR Green I and Mg^2+^ was specific used for the detection of target sequences. Each reaction mixture consists of 10μL 2 × SGExcel FastSYBR Mixture, 0.4μL Forward primer and Reverse primer (both are 10uM), 0.8 μL DNA template and 8.8uL RNase-Free ddH_2_O. The reaction program of QPCR was set as two-step thermal cycling procedure, which comprises predenaturation step at 95°C for 20s, followed by 40 cycle of 3s at 95°C for denaturation and 30s at 60°C for holding. The final step was general ending process.

Table S1 Characteristics of primers

| ID | Primer | Sequences (5'to3') |
| --- | --- | --- |
| *Archaea*  ARC | F: ARC787F | ATTAGATACCCSBGTAGTCC |
|  | R: ARC1059R | GCCATGCACCWCCTCT |
| *Methanobacteriales*  MBT | F: MBT857F | CGWAGGGAAGCTGTTAAGT |
|  | R: MBT1196R | TACCGTCGTCCACTCCTT |
| *Methanomicrobiales*  MMB | F: MMB282F | ATCGRTACGGGTTGTGGG |
|  | R: MMB832R | CACCTAACGCRCATHGTTTAC |
| *Methanosarcinaceae*  Msc | F: Msc380F | GAAACCGYGATAAGGGGA |
|  | R: Msc828R | TAGCGARCATCGTTTACG |
| *Methanosaetaceae*  Mst | F: Mst702F | TAATCCTYGARGGACCACCA |
|  | R: Mst862R | CCTACGGCACCRACMAC |
| *Bacteria*  BAC | F: BAC338F | ACTCCTACGGGAGGCAG |
|  | R: BAC805R | GACTACCAGGGTATCTAATCC |

Figure S1 Current variation within one batch

Figure S2, the equivalent circuit of EIS results

Table S2 The equivalent circuit of EIS for cathode

| Ω | R1 | R2 | R3 |
| --- | --- | --- | --- |
| 1.7mA | 2.994 | 0.7198 | 120.6 |
| 6.9mA | 3.667 | 4.772 | 93.48 |
